# *Strongyloides stercoralis* and *Trypanosoma cruzi* coinfections in a highly endemic area in Argentina

**DOI:** 10.1371/journal.pntd.0010179

**Published:** 2022-02-04

**Authors:** Pedro E. Fleitas, Noelia Floridia-Yapur, Elvia E. Nieves, Adriana Echazu, Paola A. Vargas, Nicolás R. Caro, Ramiro Aveldaño, Walter Lopez, Mariana Fernandez, Favio Crudo, Rubén O. Cimino, Alejandro J. Krolewiecki

**Affiliations:** 1 Universidad Nacional de Salta. Sede Regional Orán. Instituto de Investigación de Enfermedades Tropicales (IIET), Salta, Argentina; 2 Universidad Nacional de Salta, Facultad de Ciencias Naturales, Cátedra de Química Biológica, Salta, Argentina; 3 Consejo Nacional de Investigaciones Científicas y Técnicas (CONICET), Buenos Aires, Argentina; 4 Instituto de Patología Experimental, Consejo Nacional de Investigaciones Científicas y Técnicas (CONICET), Salta, Argentina; 5 Universidad Nacional de Salta, Facultad de Ciencias de la Salud, Cátedra de Parasitología, Salta, Argentina; 6 Biotecnología, Universidad Católica de Boliviana San Pablo, Santa Cruz, Bolivia; 7 Asociación para el Desarrollo Sanitario Regional (ADESAR), San Antonio de Areco, Buenos Aires, Argentina; 8 Fundación Mundo Sano, Buenos Aires, Argentina; 9 Universidad Nacional de San Antonio de Areco, San Antonio de Areco, Buenos Aires, Argentina; George Washington University School of Medicine and Health Sciences, UNITED STATES

## Abstract

**Background:**

Strongyloidiasis and Chagas disease are endemic in northern Argentina. In this study we evaluate the association between *S*. *stercoralis* and *T*. *cruzi* infections in villages with diverse prevalence levels for these parasites. Further understanding in the relationship between these Neglected Tropical Diseases of South America is relevant for the design of integrated control measures as well as exploring potential biologic interactions.

**Methodology:**

Community based cross-sectional studies were carried in different villages of the Chaco and Yungas regions in Argentina. Individuals were diagnosed by serology for *S*. *stercoralis* and *T*. *cruzi*. The association between *S*. *stercoralis* and *T*. *cruzi*, and between anemia and the two parasites was evaluated using two approaches: marginal (Ma) and multilevel regression (Mu).

**Results:**

A total of 706 individuals from six villages of northern Argentina were included. A total of 37% were positive for *S*. *stercoralis*, 14% were positive for *T*. *cruzi* and 5% were positive for both. No association was found between infection with *S*. *stercoralis* and *T*. *cruzi* in any of the models, but we found a negative correlation between the prevalence of these species in the different villages (r = -0.91). Adults (> 15 years) presented association with *S*. *stercoralis* (Ma OR = 2.72; Mu OR = 2.84) and *T*. *cruzi* (Ma OR = 5.12; Mu OR = 5.48). Also, 12% and 2% of the variance of infection with *S*. *stercoralis* and *T*. *cruzi*, respectively, could be explained by differences among villages. On the other hand, anemia was associated with infection with *S*. *stercoralis (*Ma OR = 1.73; Mu OR = 1.78) and was more prevalent in adults (Ma OR = 2.59; Mu OR = 2.69).

**Conclusion:**

We found that coinfection between *S*. *stercoralis* and *T*. *cruzi* is not more frequent than chance in endemic areas. However, the high prevalence for both parasites, raises the need for an integrated strategy for the control of STH and Chagas disease.

## Author summary

Infections with *S*. *stercoralis* and *T*. *cruzi* belong to the group of neglected tropical diseases (NTDs). NTDs are influenced by climate and the presence of their vectors and host reservoirs in areas with structural poverty. *S*. *stercoralis* and *T*. *cruzi* have high prevalence in northern Argentina. Due to the high prevalence and chronicity of infections by these parasites, coinfection with both is possible. We conducted a cross sectional study in six villages in the north of Argentina. These villages have different prevalence for the two parasites and are located in different ecoregions (Chaco, Yungas and the border between these two). We found that coinfection between *S*. *stercoralis* and *T*. *cruzi* occurs only by chance; and the most affected groups for *S*. *stercoralis* and *T*. *cruzi* infection are adults. We also found that adults or individual with infection with *S*. *stercoralis* were more likely to have anemia. In addition, each village represents a different setting for infection with *S*. *stercoralis* and *T*. *cruzi*, and individuals in different villages presented differences in the probability of infection.

## Introduction

Neglected tropical diseases (NTDs) include a group of infectious diseases that have a significant impact on individuals, families, and communities in developing countries in terms of disease burden, quality of life, productivity loss and poverty aggravation as well as the high cost of long-term care [1]. Neglected tropical diseases are not widely distributed; they are restricted by climate and the presence of their vectors and host reservoirs in areas with structural poverty [1]. Soil-transmitted helminths (STH) and Chagas disease are both NTDs.

*Strongyloides stercoralis* is a STH with a complex life cycle and the ability to reproduce in soil and within the human host. The infection occurs by larvae that penetrate the skin; these larvae are found in soil contaminated with feces. However, unlike other STH, *S*. *stercoralis* has the capacity for autoinfection, and generates a chronic infection that can last for decades.[2,3]. While not yet included in most STH control programs, the recently issued list of targets for STH control programs by the World Health Organization (WHO) includes the establishment of an efficient strongyloidiasis control program in school age children (SAC) by 2030 [4]. It is estimated that there are 386 million people infected by *S*. *stercoralis* worldwide [5].

Chagas disease is caused by the protozoan *Trypanosoma cruzi*. The infection can occur in three major ways. A vector pathway (feces of infected triatomines come into contact with the mucous membranes or skin lesions), a congenital pathway (parasites infect the fetus during pregnancy) and by organ or blood transfusion. After acute infection, chronic infection continues for life. Although most remain in the chronic phase without symptoms, between 30% and 40% of them will develop clinical cardiac and/or digestive manifestations [6,7]. It is estimated that there are 6–7 million people infected by *T*. *cruzi* worldwide [8].

*S*. *stercoralis* and *T*. *cruzi* are endemic in northern Argentina [9,10], along with other STH and *Leishmania* [11,12]. In this region, there is a high prevalence of the Chagas disease vector, *Triatoma infestans*, with a prevalence of *T*. *cruzi* that can exceed 50% in localized communities [13,14]. In addition, northern Argentina has varied prevalence of *S*. *stercoralis* ranging from 7% to over 40% [9]. Due to the high prevalence and the long duration of infections by these parasites, coinfection with both is possible. Coinfection studies have focused on similar parasites or those that produce a dramatic effect (HIV); however, there is a lack of knowledge about the interactions between common parasites [15]. In addition, co-infection with different NTDs is a risk in human populations living in resource-limited settings, and an association between *S*. *stercoralis* and *T*. *cruzi* has been found among migrants from Latin America in non-endemic areas [16]. Therefore, for a better understanding of the epidemiology of these parasites, it is necessary to know if there is an association between *S*. *stercoralis* and *T*. *cruzi* in endemic areas for both, or if coinfections occur only by chance.

## Methods

### Ethical statement

Ethical approval was obtained from the Bioethics Committee of College of Physicians of Salta Province, Argentina (N°14.200), and the Faculty of Health Sciences from the Universidad Nacional de Salta. The study was conducted in accordance with principles of the 2013 Declaration of Helsinki. Sera samples collected in each survey were treated according to the study protocols approved of each particular study. Participation was voluntary, and written informed consent was obtained from all patients involved in the study. For child participants, written informed consent was obtained from the parent or guardian.

### Objectives

The main aim of this study was to evaluate the association between *S*. *stercoralis* and *T*. *cruzi* infections in different endemic areas located in northern Argentina. As a secondary objective, the association between anemia and *S*. *stercoralis* and *T*. *cruzi* infections was evaluated.

### Study population and description of the geographical areas

**Fig 1:**
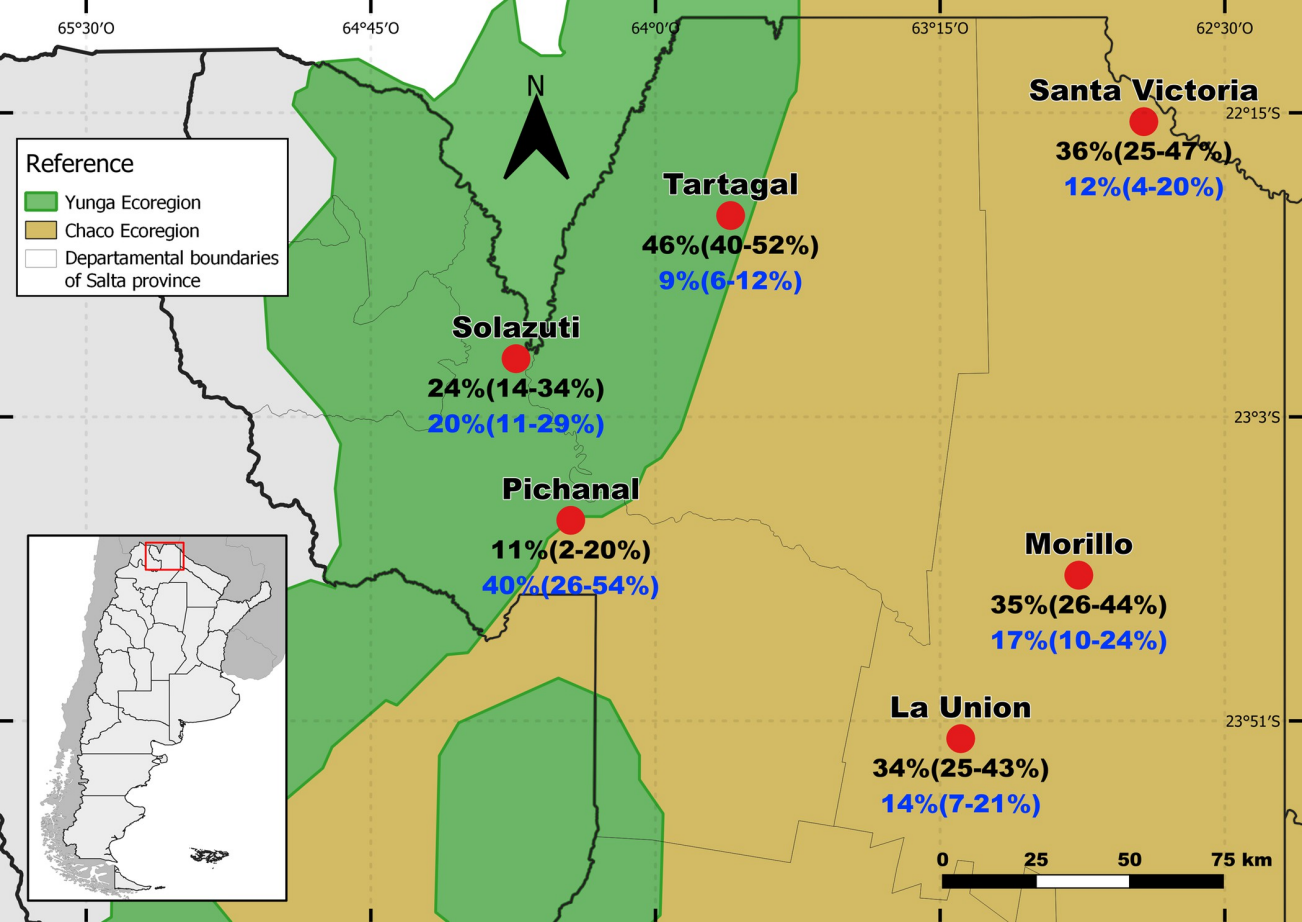
Villages and ecoregions where the samples were collected. The prevalence (95% CI) of *S*. *stercoralis* (black) and *T*. *cruzi* (blue) is shown for each village. This figure was created using free and freely available shapefiles [19–21].

Community based cross-sectional studies were carried out from 2010 until 2016 in different villages of the Chaco and Yungas regions in Argentina, towards evaluating the prevalence and distribution of different NTDs [9,17,18]. Sampled individuals were randomly selected from a population-based census using either individuals or households as units for randomization. Venous blood was extracted from all the individuals, from the different villages, who participated in the different studies for diagnostic analysis. Villages with serological data for only one of the parasites (*S*. *stercoralis* or *T*. *cruzi*) were not included in this study. This study included 706 individuals from six villages in the province of Salta, in northwestern Argentina, who had a complete serological data for the two parasites obtained from epidemiological surveys [9,17,18]. One village is located in the Yungas ecoregion (Solazuti), three in the Chaco ecoregion (Santa Victoria, Morillo and La Union), and two on the border between the two (Tartagal and Pichanal) (Fig 1).

The dry Chaco ecoregion in Argentina is a vast plain, which has large daily temperature amplitude associated with seasonal variation. In winter, the entry of cold fronts causes frosts throughout the ecoregion. In turn, the Yungas ecoregion [22] has a subtropical climate with rainfalls concentrated in the summer period (November-March). The topography of these regions leads the winds to rise and discharge the humidity as the altitude increases, which results in lower temperatures in elevated areas [22].

### Serological tests for *Strongyloides stercoralis* and *Trypanosoma cruzi*

All participants in the study had 5 mL of blood drawn by venipuncture. A complete blood count was performed with a SYSMEX KX 21N automated hematology analyzer. Hemoglobin (Hgb) value results were recorded and individuals were classified as anemic or non-anemic using the Hgb thresholds for defining anemia according to sex and age established by WHO/UNICEF [23]. In addition, blood samples were centrifuged and an aliquot of serum was preserved frozen at -20°C for subsequent serological diagnosis of *S*. *stercoralis* and *T*. *cruzi*.

The diagnosis of *S*. *stercoralis* was performed by in-house enzyme-linked immunosorbent assay (NIE-ELISA) method. NIE-ELISA detects IgG antibodies against the recombinant protein NIE of *S*. *stercoralis* as described previously [24]. This method presented a sensitivity and specificity of 75.4% and 94.8% in a blinded study [25], and it does not cross-react with other helminths such as *Ascaris lumbricoides* or hookworms [25,26]. Patient’s sera were tested in duplicate and compared to a standard positive IgG curve run on each plate. The averages of duplicate results were calculated and corrected for background reactivity (no serum added). Seropositive subjects for *S*. *stercoralis* infection had IgG titers against NIE antigen above the selected cutoff value. These subjects were entered in a database as cases.

The diagnosis of *T*. *cruzi* was performed by ELISA and indirect hemagglutination assay (IHA) with commercial kits (Chagatest ELISA and Chagatest HAI, Wiener Lab, Argentina). Specimens were tested in duplicate by both tests. In case of disagreement between the two techniques, indirect immunofluorescence was performed (Inmunofluor CHAGAS, BIOCIENTIFICA, Argentina).

### Statistical analysis

All statistical analyses were performed with R software [27]. For the marginal models, and the respective odd ratios with the 95% confidence interval, the geepack library was used; and the village was used as a cluster with the exchangeable working correlation matrix [28]. For the multilevel models, and the respective odd ratios with the 95% confidence interval, the lme4 library was used, and the village was used as a random effect [29]. In addition, the Intra-class correlation coefficient (ICC) and the median odds ratio (MOR) were calculated [30].

Categorical variables were described by counts and percentages, and chi-square Pearson test was used to compare the distribution. In addition, the relationship between the prevalence of *S*. *stercoralis* and *T*. *cruzi*, was analyzed by Pearson´s correlation and linear regression, weighted by the sample-size in each village.

Association between infection by *S*. *stercoralis* and *T*. *cruzi*, and between anemia and the two parasites was analyzed by regression analysis. The data was considered correlated because individuals in a village share a similar state of health between each other than from outer populations, therefore they share a series of environmental, economic, social and other characteristics [30]. Two different approaches to data analysis were addressed, marginal and multilevel regression [31,32]. The marginal models estimate population-averaged (or population-marginalized) parameters [33]. In this marginal model, the effects of the covariates are inferred at the group level. Therefore, the odds ratios (OR) characterize the effect of covariates in the population as a whole, rather than in a particular village. In the multilevel model, the effects of the covariates are inferred at the individual level. Thus, for individual-level variables, the OR interpretations apply for comparisons of individuals within the same village.

The association of the infection of *S*. *stercoralis* with age, sex and infection with *T*. *cruzi* was analyzed, as well as the association of anemia with age, sex, infection with *T*. *cruzi* and infection with *S*. *stercoralis*. Age was considered as a dichotomous variable as children≤ 15 years and adults > 15 years.

## Results

### Study population characteristics

The total prevalence of *S*. *stercoralis*, *T*. *cruzi* and co-infection was 37%, 14% and 5%, respectively. There were no differences in prevalence seen in the *T*. *cruzi* infected population and presence or absence of *S*. *stercoralis* infection (Table 1). The highest prevalence of *S*. *stercoralis* was observed in adults (45%) and in males (34%), while the highest prevalence of *T*. *cruzi* was observed in adults (70%) regardless of sex.

**Table 1:** Characteristics of the individuals included in the analysis.

**Baseline characteristics of 706 individuals included in the study, according to results on *T*. *cruzi* serology, *S*. *stercoralis* serology and Anemia**									
**Variable**	***T*. *cruzi* positive (n = 101)**	***T*. *cruzi* negative (n = 605)**	**p**	***S*. *stercoralis* positive (n = 261)**	***S*. *stercoralis* negative (n = 445)**	**p**	**Anemia present (n = 287)**	**Anemia absent (n = 419)**	**p**
**Male**	44 (44%)	234 (39%)	0.35	89 (34%)	189 (43%)	0.03	104 (34%)	174 (42%)	0.15
**Adult**	71 (70%)	189 (31%)	<0.01	118 (45%)	142 (32%)	<0.01	150 (52%)	110 (26%)	<0.01
***T*. *cruzi positive***	-----------	-----------	-------	34 (13%)	67 (15%)	0.45	53 (19%)	48 (11%)	0.01
***S*. *stercoralis* positive**	34 (34%)	227 (38%)	0.45	--------	-----------	-------	131 (46%)	130 (31%)	<0.01

**Characteristics of the population of each village**										
**Village**	**n (** * **Popu-lation)**	**Sex**	**%**	**Age**				**Prevalence of *S*. *stercoralis* (CI 95%)**	**Prevalence of *T*. *cruzi* (CI 95%))**	**Anemia (%)**
					**%**	**Median**	**IQR**			
**La Union**	106 (2353)	Female	61	Children	48	8	(11–5)	34 (25–43)	14 (7–21)	29
		Male	39	Adults	52	38	(49–23)			
**Morillo**	106 (4714)	Female	57	Children	72	7	(10–4)	35 (26–44)	17 (10–24)	35
		Male	43	Adults	28	30	(38–23)			
**Pichanal**	45 (2311)	Female	53	Children	31	9	(12–4)	11 (2–20)	40 (26–54)	36
		Male	47	Adults	69	33	(49–22)			
**Solazuty**	76 (308)	Female	54	Children	34	8	(12–5)	24 (26–54)	20 (11–29)	75
		Male	46	Adults	66	34	(53–23)			
**Santa Victoria**	67 (1809)	Female	72	Children	62	5	(8–3)	36 (25–47)	12 (4–20)	42
		Male	28	Adults	38	28	(33–19)			
**Tartagal**	306 (5155)	Female	62	Children	78	9	(11–6)	46 (40–52)	9 (6–12)	39
		Male	38	Adults	22	32	(40–22)			

* The population corresponds to the census carried out by the primary health care system at the time of each study.

The age range of children (0 to 14 years-old) and adults (≥15 years-old) was similar in all villages (Table 1). The village of Solazuti presented a significantly higher prevalence of anemia (75%) than the other villages. The village with the highest prevalence of *S*. *stercoralis* was Tartagal (46%), coinciding with the lowest prevalence of *T*. *cruzi* (9%). The village with the highest prevalence of *T*. *cruzi* was Pichanal (40%), coinciding with the lowest prevalence of *S*. *stercoralis* (11%). In reference to this, we found a strong negative correlation between the prevalence of *S*. *stercoralis* and the prevalence of *T*. *cruzi* in the different villages (r = -0.91, r^2^ = 0.81, p = 0.01).

### Association between *S*. *stercoralis* and *T*. *cruzi* infections

No association was found between infection with *S*. *stercoralis* and *T*. *cruzi* in any of the models (Tables 2 and 3). Infection with *S*. *stercoralis* showed an association only with age in both models (Table 2). According to the marginal regression (OR = 2.72), on average, adults have 2.72 higher odds than children to be infected with *S*. *stercoralis*. On the other hand, the multilevel regression odds ratio of 2.84 for age, describes that between two individuals with identical risk factors, a child and an adult, and with identical environmental characteristics with respect to the risk of infection with *S*. *stercoralis*, then the probabilities of being infected increased 2.84 folds for the adult. In addition, in the marginal model the correlation parameter between *S*. *stercoralis* infection outcomes for any two individuals from the same village was 0.11, suggesting a weak positive association. The multilevel model estimated that 12% of variance (ICC = 0.12) of infection with *S*. *stercoralis* can be explained by the differences among villages. In addition, if two individuals with the same characteristics in different villages are compared, there would be a median increase in the odds of infection of 1.89 (MOR = 1.89).

**Table 2:** Marginal and multilevel logistic regression that describes associations between *S*. *stercoralis* infection and age, sex, and infection with *T*. *cruzi*.

Variable	Marginal*			Multilevel**		
	OR	95% CI	p	OR	95% CI	p
Sex (Male)	0.80	0.56–1.16	0.228	0.80	0.57–1.11	0.181
Age (Adult)	2.72	1.16–6.39	0.022	2.84	1.94–4.15	<0.001
Infection with *T*. *cruzi* (positive)	0.77	0.45–1.31	0.335	0.78	0.46–1.23	0.255

*Correlation parameter: estimate = 0.11, Std.err = 0.04

** Random effect: variance = 0.447, Std.Dev = 0.668, ICC = 0.12 MOR = 1.89

**Table 3:** Marginal and multilevel logistic regression that describes associations between *T*. *cruzi* infection and age, sex, and infection with *S*. *stercoralis*.

Variable	Marginal*			Multilevel**		
	OR	95% CI	p	OR	95% CI	p
Sex (Male)	1.38	1.00–1.91	0.05	1.42	0.89–2.23	0.140
Age (Adult)	5.12	2.72–9.62	<0.001	5.48	3.34–9.023	<0.001
Infection with *S*. *stercoralis* (positive)	0.73	0.43–1.23	0.24	0.73	0.44–1.20	0.210

*Correlation parameter: estimate = 0.01, Std.err = 0.02

** Random effect: variance = 0.0806, Std.Dev = 0.284, ICC = 0.02 MOR = 1.31

Infection with *T*. *cruzi* showed an association with age in both models (Table 3), on average adults have 5.12 higher odds than children, and at the individual level adults have 5.48 higher odds than children. In addition, the multilevel model estimated that 2% of variance (ICC = 0.02) of infection with *T*. *cruzi* can be explained by the differences between villages; and If two individuals with the same characteristics in different villages are compared, there would be a median increase in the odds of infection of 1.31 (MOR = 1.31).

### Association between anemia and infection with *S*. *stercoralis* and *T*. *cruzi*

The cases of anemia were associated with age and infection with *S*. *stercoralis* in both models (Table 4). In turn, the multilevel model estimated that 10% of variance (ICC = 0.10) of anemia cases can be explained by the differences between villages. In addition, if an individual moves to a location with a higher probability of anemia, there would be a median increase in the odds of 1.77 (MOR = 1.77).

**Table 4:** Marginal and multilevel logistic regression that describes associations between anemia and age, sex, and infection with *S*. *stercoralis* and *T*. *cruzi*.

Variable	Marginal*				Multilevel**		
	OR		95% CI	p	OR	95% CI	p
Sex (Male)	0.88	0.70–1.10		0.248	0.87	0.62–1.22	0.422
Age (Adult)	2.59	1.62–4.16		<0.001	2.69	1.86–3.89	<0.001
Infection with *T*. *cruzi* (positive)	1.27	0.99–1.63		0.063	1.31	0.81–2.12	0.273
Infection with S. stercoralis (positive)	1.73	1.26–2.38		<0.001	1.78	1.26–2.51	0.001

* Correlation parameter: estimate = 0.02, Std.err = 0.03

**Random effect, variance = 0.357, Std.Dev = 0.597, ICC = 0.10, MOR = 1.77

On average, individuals infected with *S*. *stercoralis* have 1.73 higher odds to have anemia than those not infected. In addition, two individuals with identical risk factors, one infected with *S*. *stercoralis* and the other not, and with identical characteristics regarding the risk of having anemia, then the probabilities of having anemia increased 1.78 folds for those infected with *S*. *stercoralis*.

## Discussion

Northern Argentina has been identified as a hot spot for NTDs [34]. Chagas disease and STH, including *S*. *stercoralis*, are among the main NTDs with active transmission in the region [35,36]. Regarding Chagas disease, northern Argentina has high prevalence, where vector transmission persists as a consequence of home infestation with *T*. *infestans*, the main vector in the area [13,14]. In addition, previous studies showed the high prevalence *of S*. *stercoralis* in the same area [9]. In this cross-sectional study in six villages, we analyze the possible association between *S*. *stercoralis* and *T*. *cruzi*.

Recent studies in non-endemic areas have shown a prevalence of more than 20% for coinfection with *S*. *stercoralis* and *T*. *cruzi* in migrants from Latin America [16,37]; and >10% coinfection in populations with chronic Chagas disease [38,39]. In addition, studies carried out in non-endemic areas showed association after adjusting for sex, age and country of origin [16]. However, in our study, carried out in endemic areas of Argentina, where 5% were co-infected with *S*. *stercoralis* and *T*. *cruzi*, we found no association between them. This is not surprising considering that although both infections are prevalent in the area, they do not share transmission paths, and their biological cycles are different. However, the main socioeconomic risk factors associated with STH (water, sanitation and overcrowding) and *T*. *cruzi* (water, adobe houses and wood floor) coincide in places of extreme poverty where high prevalence are registered for these parasites [40,41]. Therefore, coinfection may be linked to poverty rather than to biological factors. Nevertheless, since both parasites have overlapping distribution and produce silent infections, the active search for susceptible population for both is a rational strategy for an integrated management of infected individuals [37]. In addition, although the prevalence of coinfection may be low, the immune response to *S*. *stercoralis* might affect the response to *T*. *cruzi* and affect the evolution of Chagas disease [42].

It should be noted that the place of origin, as reported by other authors [16,37], plays a role in the probability of infection. This role was more marked in infections with *S*. *stercoralis*, where a slight correlation was found between individuals from the same village, and 12% of the variability of prevalence of infection could be explained by differences between the villages. This implies that each village represents a different setting for infection with *S*. *stercoralis*, and individuals in different villages presented differences in the probability of infection. In addition, the variability of the infection between the different villages may be due to differences in annual temperatures and precipitations, which are the main environmental factors linked to STH [40]. On the other hand, only 2% of the variability of infection with *T*. *cruzi* could be explained by differences among villages. This low prevalence may be due to the fact that altitude has been proposed as the main environmental variable associated with *T*. *cruzi* infection [41], and the villages analyzed in this study have similar altitudes.

Regression models for infection with *S*. *stercoralis* showed an association with age. This is in agreement with other reports, where adults have a higher risk of infection than children [43]. This association is because *S*. *stercoralis* infection can occur at a young age and remain for decades in the host if untreated, leading to an accumulation of infection over time [43]. On the other hand, no association with sex was found, and the significant difference between the percentages of men among those infected with *S*. *stercoralis* is due to the fact that age acts as a confounding variable. Although studies conducted in Laos and Cambodia reported that men had a higher risk of infection [43]. This discrepancy between Argentina and Laos with respect to the prevalence of *S*. *stercoralis* in males may be due to cultural differences associated with the practice of agriculture. In Laos, there is a higher prevalence of *S*. *stercoralis* in males because farmers work without shoes, while females restrict their work activities to the home and wear shoes [44].

Regression models for infection with *T*. *cruzi* showed an association with age. Adults have > 5 odds to be infected than children. Regarding this, it has been previously reported in areas of Argentina, a higher prevalence in those over 30 years of age and lower prevalence in children under five years of age [14]. This highlights that the strategies for the control of Chagas disease that have been developed for the last 40 years in the country produced a reduction in the incidence of the disease [45], but an elimination of the infection in children has not yet been achieved, highlighting the need for action against vertical transmission of Chagas disease through screening and treatment of women and infants [46].

Although no association was found between infection with *S*. *stercoralis* and *T*. *cruzi*, we found a negative correlation between the prevalence of *S*. *stercoralis* and *T*. *cruzi* in the different villages located in the Chaco and Yungas ecoregions which needs to be confirmed in larger studies and occurs in a context of high prevalence of both infections. This could imply the existence of variables that produce different effects on these parasites. However, this could not be analyzed in this study.

The prevalence of anemia in the different villages make them of severe public health significance as defined by WHO [23]. The prevalence of iron deficiency varies greatly depending on host factors: age, gender, physiological, pathological, environmental and socioeconomic terms [23]. In the cases of anemia analyzed in this study, in an area where frequent causes of anemia in the tropics as malaria and schistosomiasis are absent, it was found that 10% of the variability was due to differences between villages. In addition, the cases of anemia were associated infection with *S*. *stercoralis*. This is in accordance with what was reported in Tartagal, where not only an association between anemia and *S*. *stercoralis* was found, but also the hematological parameters improved after deworming [18]. Anemia was also associated with age, with adults being more likely to be anemic than children. This is due to the fact that many of the children attend school canteens, where there is a balanced diet, and there are anemia control programs in the child population, while the adult population is a neglected population. On the other hand, no association was found between anemia and infection with *T*. *cruzi*. This is to be expected since cases of anemia have only been reported in individuals with reactivation of infection with *T*. *cruzi*, and not in chronic cases [47]. In addition, the significant difference between the percentage of infected with *T*. *cruzi* among cases of anemia is due to the fact that infection with *T*. *cruzi* is associated with age, and age is associated with anemia.

As could be observed in this study, the coefficients of the different marginal and multilevel regression models were almost the same. This is due to the low intra-village association, translated into correlation coefficients close to zero in the marginal models and a low variance of the random effect in the multilevel model. While the models for *S*. *stercoralis* and Anemia showed a slight correlation of the data, the models for *T*. *cruzi* showed a very low correlation. However, ignoring the correlation present in the data, as occurs in ordinary logistic regression, results in an overestimation of the coefficients. In addition, although the value of the coefficients is similar in the multilevel and marginal models, the meaning is different and the use of one model instead of the other depends fundamentally on the objectives to be achieved. If the objective is to measure the effect of the covariates for a given village, the multilevel model is recommended; on the other hand, if the objective is to measure the global response of the covariates adjusted for the heterogeneity of the villages, the marginal model is recommended [30].

Some limitations must be taken into account to interpret this study. First, estimating OR from cross-sectional data is of somewhat limited value, because while ORs can discover associations, they generally impede to elucidate causality. Second, the serological diagnosis of *S*. *stercoralis* fails to differentiate between active or past infection [17,48]. However, there was no deworming treatment for *S*. *stercoralis* in the different villages at baseline, so it can be assumed that infections were active. In addition, serological techniques have demonstrated their validity and feasibility in epidemiological surveys, allowing the possibility of processing a large number of samples for different pathogens [9]. Third, epidemiological information from the different villages is incomplete. All the villages have adobe houses, overcrowding, lack of access to drinking water and sanitation, but the percentage of these factors is unknown, so it could not be evaluated in this study. Four, the villages have high prevalence of hookworms, and hookworms are associated with anemia and infection with *S*. *stercoralis* [5]; Therefore, it is plausible that the association between *S*. *stercoralis* and anemia is due in part to hookworms. Further studies are needed in the different villages to measure the prevalence of hookworm, levels of malnutrition, and prevalence of anemia-causing diseases in order to have a better understanding of the high prevalence of anemia. Five, although biases in the sampling cannot be ruled out, the prevalence of infected reported in this work are within the confidence interval of the prevalence reported in the area [9]. Finally, this study is not intended to discuss on a clinical basis. This study represents a first approach to the epidemiology of *S. stercoralis* and *T*. *cruzi* coinfection in endemic areas in order to explore the possibility for integration of control activities as well as identifying high risk areas.

In summary, we found that coinfection between *S*. *stercoralis* and *T*. *cruzi* is not more frequent than chance in endemic areas. However, the high prevalence for both parasites raises the need for an integrated strategy for the control of STH and Chagas disease.
